# Supplementary material for: Development of a Monoclonal Antibody Targeting HTLV-1 Envelope gp46 Glycoprotein and Its Application to Near-Infrared Photoimmuno-Antimicrobial Strategy
Source: Viruses. 2022 Sep 29;14(10):2153. doi: 10.3390/v14102153 (PMC9608601; doi:10.3390/v14102153)
Supplement: Supplementary file 1 [file viruses-14-02153-s001.zip › viruses-1840742-supplementary.pdf]

## Supplementary Materials

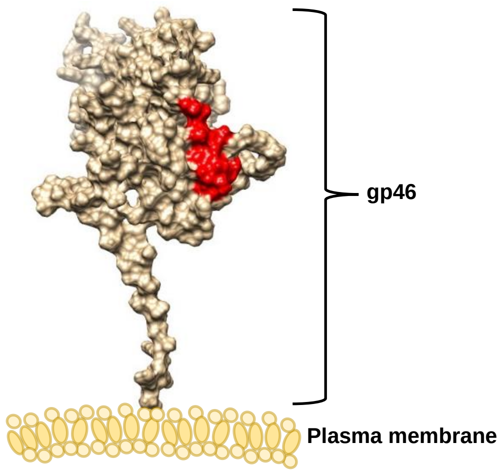


**Figure S1**. Position of the mAb Clone D epitope sequence (highlighted in red) in a predicted structural model of HTLV-1 gp46 protein monomer.

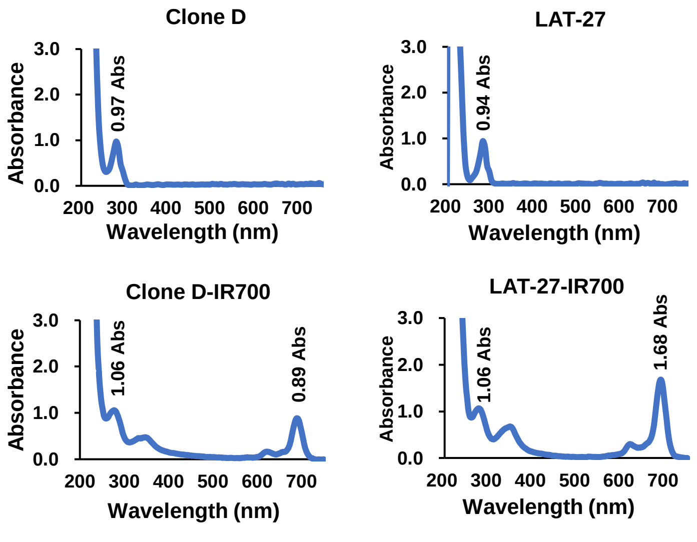


**Figure S2.** Confirmation of IR700 conjugation efficiency on antibodies intended for use in near-infrared photoimmuno-antimicrobial strategy. Concentrations of antibody and IR700 were determined by absorbance measurement at 280 nm and 689 nm. The mAb bound to the phthalocyanine dye IR700 showed photosensitivity to near-infrared irradiation.

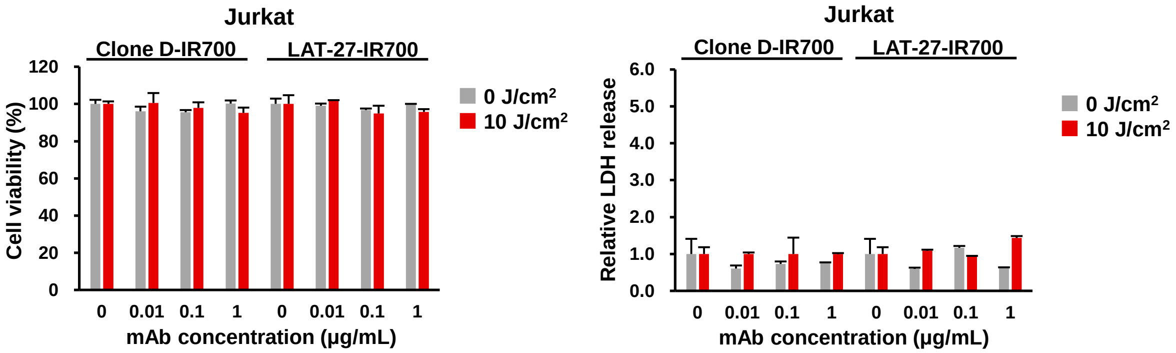


**Figure S3.** Results of cell viability assays (left) and lactate dehydrogenase (LDH) assays (right) using Clone D-IR700 and LAT-27-IR700 for Jurkat cells. Cell viability assays show the percentage of cell viability is 100% at 0 J/cm$^2$ and an mAb concentration of 0 μg/mL. LDH assays show relative LDH release normalized at 0 J/cm$^2$ and mAb concentration of 0 μg/mL. Error bars represent the SD of triplicate testing.

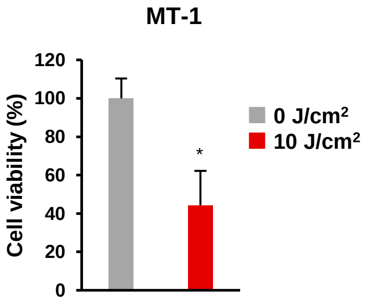


**Figure S4.** Results of cell viability assays using Clone D-IR700 (1 μg/mL) for MT-1 cells irradiated (10 J/cm$^2$) or non-irradiated (0 J/cm$^2$) with near infrared light. Error bars represent the SD of triplicate testing. Significance is indicated by *p*-values, as follows: $^*p < 0.01$.

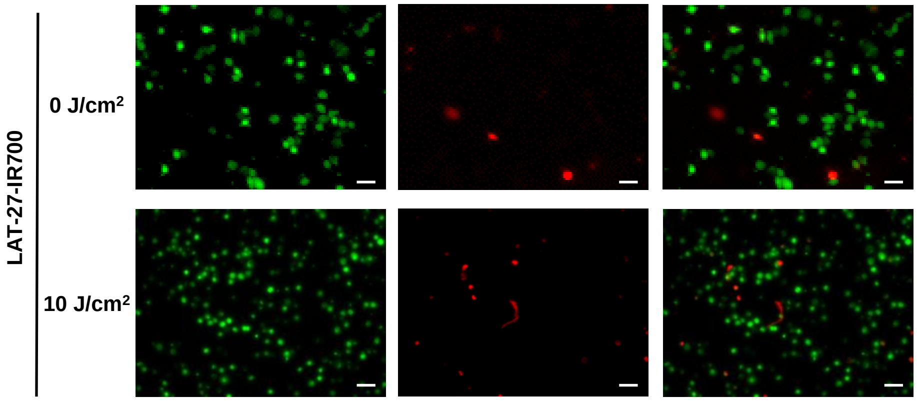


**Figure S5.** Cell staining of MT-2 cells irradiated (10 J/cm$^2$) or not irradiated (0 J/cm$^2$) with near-infrared light after incubation with LAT-27-IR700. Live cells were stained with Calcein (green) and dead cells with EthD-III (red). Scale bar = 20 μm.
